# P-2191. Efficacy and safety of buleviritide monotherapy for chronic hepatitis D in patients with and without cirrhosis: results from the week 144 interim analysis of a phase 3 randomized study

**DOI:** 10.1093/ofid/ofae631.2345

**Published:** 2025-01-29

**Authors:** Soo Aleman, Heiner Wedemeyer, Maurizia Brunetto, Antje Blank, Pietro Andreone, Pavel Bogomolov, Vladimir Chulanov, Nina Mamonova, Natalia Geyvandova, Viacheslav Morozov, Olga Sagalova, Tatyana Stepanova, Grace Chee, Dmitry Manuilov, Mingyang Li, Audrey Lau, Anu Osinusi, Steve Tseng, Julian Schulze Zur Wiesch, Markus Cornberg, Stefan Zeuzem, Pietro Lampertico

**Affiliations:** Department of Infectious Diseases, Karolinska University Hospital/Karolinska Institutet, Stockholm, Sweden, Stockholm, Sodermanlands Lan, Sweden; Clinic for Gastroenterology, Hepatology, Infectious Diseases and Endocrinology, Hannover Medical School, Hannover, Germany, Hannover, Niedersachsen, Germany; Hepatology Unit, Reference Center of the Tuscany Region for Chronic Liver Disease and Cancer, University Hospital of Pisa, Pisa, Italy; Department of Clinical and Experimental Medicine, University of Pisa, Pisa, Italy, Pisa, Toscana, Italy; Medical Faculty Heidelberg/Heidelberg University Hospital, Department of Clinical Pharmacology and Pharmacoepidemiology, Heidelberg University Hospital, Heidelberg, Germany, Heidelberg, Baden-Wurttemberg, Germany; University of Modena and Reggio Emilia, Internal Medicine, Baggiovara Hospital, Modena, Emilia-Romagna, Italy; M.F. Vladimirsky Moscow Regional Research and Clinical Institute, Moscow, Russian Federation, Moscow, Moskva, Russia; Sechenov University, Moscow, Moskva, Russia; FSBI National Research Medical Center for Phthisiopulmonology and Infectious Diseases of the Ministry of Health of the Russian Federation, Moscow, Russian Federation, Moscow, Moskva, Russia; Stavropol Regional Hospital, Stavropol, Stavropol', Russia; LLC Medical Company Hepatolog, Samara, Russian Federation, Samara, Samara, Russia; South Ural State Medical University, Chelyabinsk, Russian Federation, Chelyabinsk, Moskva, Russia; LLC Clinic of Modern Medicine, Moscow, Russian Federation, Moscow, Moskva, Russia; Gilead Sciences, Inc., Foster City, California; Gilead Sciences, Inc., Foster City, California; Gilead Sciences, Inc., Foster City, California; Gilead Sciences, Inc., Foster City, California; Gilead, Foster City, California; Gilead Sciences, Inc., Foster City, California; Hepatology Outpatient Medical Clinic, University Hospital Hamburg-Eppendorf, Hamburg, Germany, Hannover, Niedersachsen, Germany; Clinic for Gastroenterology, Hepatology, Infectious Diseases and Endocrinology, Hannover Medical School, Hannover, Germany, Hannover, Niedersachsen, Germany; Department of Medicine, University Hospital Frankfurt, Frankfurt am Main, Germany, Frankfurt, Hessen, Germany; CRC “A. M. and A. Migliavacca” Center for Liver Disease, Department of Pathophysiology and Transplantation, University of Milan, Milan, Lombardia, Italy

## Abstract

**Background:**

Bulevirtide (BLV) is a first-in-class entry inhibitor for chronic hepatitis D (CHD) approved in Europe. Interim results from MYR301 (NCT03852719), a phase 3 randomized study, showed monotherapy with BLV 2 mg/d or 10 mg/d given subcutaneously was effective and safe over 144 weeks (W). With a greater risk of developing cirrhosis with HDV than HBV alone, we explore the impact of cirrhosis status on efficacy and safety of BLV monotherapy through 144W.

**Table 1.**
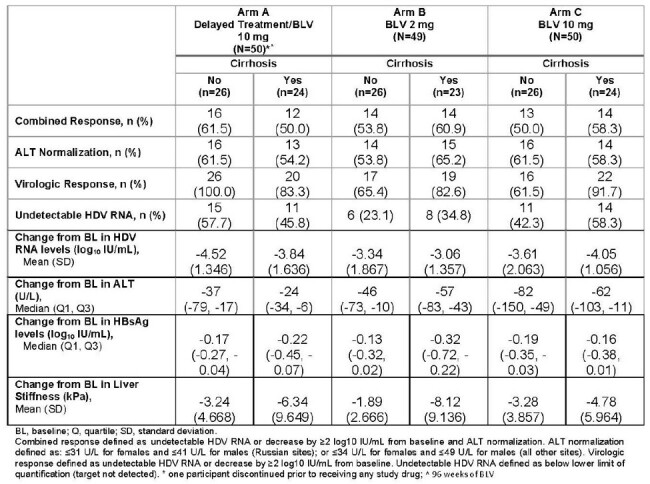
Efficacy results at Week 144 based on cirrhosis status

**Methods:**

150 patients with CHD were randomized and stratified based on the presence/absence of compensated cirrhosis (investigator-determined) as follows: Arm A: no anti-HDV treatment for 48W followed by BLV 10mg/d for 96W (n=51), Arm B: immediate treatment with BLV at 2 mg/d (n=49) or Arm C: immediate treatment with BLV at 10 mg/d (n=50) each for 144W. Combined response (CR) was defined as undetectable HDV RNA or decrease by ≥ 2 log_10_ IU/mL from baseline (virologic response (VR)) and ALT normalization. Other endpoints included VR, ALT normalization and undetectable HDV RNA. HDV RNA was quantified using RoboGene 2.0 (lower limit of quantification, 50 IU/mL; limit of detection, 6 IU/mL).

**Table 2:**
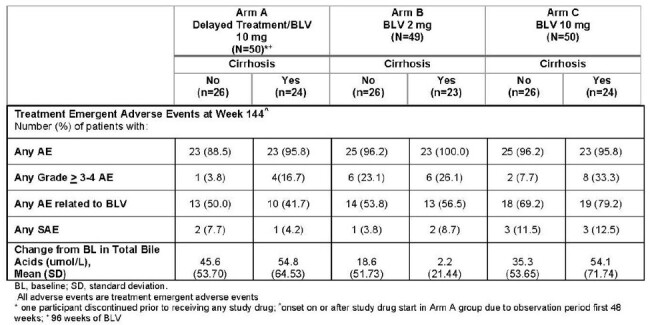
Safety results for BLV at Week 144 based on cirrhosis status

**Results:**

A total of 71/150 (47%) participants had compensated cirrhosis; 73% and 27% were Child-Pugh score 5 and 6 respectively. Baseline (BL) characteristics were similar between those with and without cirrhosis with a few expected differences; higher liver stiffness measurement (LSM) and lower platelet count in those with cirrhosis. Differences in efficacy response rates by cirrhosis status were not consistently observed (Table 1). In a univariate logistic regression in W144 completers in Arm B and C, with BL HDV RNA > 250 IU/mL, cirrhosis status was not a predictor of efficacy. Declines in both HDV RNA and ALT over time were consistent across subgroups regardless of cirrhosis status. There was no progression to liver-related outcomes over 144W except for 1 case of mild ascites in a patient with cirrhosis in Arm A. BLV was safe and well tolerated in both patients with and without cirrhosis (Table 2).

**Conclusion:**

**BLV monotherapy through Week 144 maintains efficacy and safety in patients regardless of cirrhosis status at baseline.**

**Disclosures:**

Soo Aleman, MD, PhD, AbbVie: Grant/Research Support|AbbVie: Honoraria|Biogen: Honoraria|Gilead Sciences, Inc.: Grant/Research Support|Gilead Sciences, Inc.: Honoraria|MSD: Honoraria Heiner Wedemeyer, MD, PhD, Abbott: Advisor/Consultant|Abbott: Honoraria|AbbVie: Advisor/Consultant|AbbVie: Honoraria|Arbutus: Advisor/Consultant|Boeringer Ingelheim: Advisor/Consultant|Boeringer Ingelheim: Honoraria|Bristol Myers Squibb: Advisor/Consultant|Bristol Myers Squibb: Honoraria|Dicerna: Advisor/Consultant|Gilead Sciences, Inc.: Advisor/Consultant|Gilead Sciences, Inc.: Honoraria|Johnson & Johnson/Janssen-Cilag: Advisor/Consultant|Johnson & Johnson/Janssen-Cilag: Honoraria|Merck/Schering-Plough: Advisor/Consultant|Merck/Schering-Plough: Honoraria|MYR GmbH: Advisor/Consultant|MYR GmbH: Honoraria|Novartis: Advisor/Consultant|Novartis: Honoraria|Roche: Advisor/Consultant|Roche: Honoraria|Siemens: Advisor/Consultant|Siemens: Honoraria|Transgene: Advisor/Consultant|Transgene: Honoraria|Viiv Healthcare: Advisor/Consultant|Viiv Healthcare: Honoraria|Vir Biotechnology: Advisor/Consultant Maurizia Brunetto, MD, AbbVie: Advisor/Consultant|AbbVie: speaker bureau|EISAI-MSD: Advisor/Consultant|EISAI-MSD: speaker bureau|Gilead Sciences, Inc.: Advisor/Consultant|Gilead Sciences, Inc.: speaker bureau|Janssen: Advisor/Consultant|Janssen: speaker bureau|Roche: Advisor/Consultant|Roche: speaker bureau Antje Blank, MD, Myr GMBH: Grant/Research Support Vladimir Chulanov, MD, PhD, AbbVie: Advisor/Consultant|AbbVie: Expert Testimony|AstraZeneca: Advisor/Consultant|AstraZeneca: Expert Testimony|Bristol Myers Squibb: Advisor/Consultant|Bristol Myers Squibb: Expert Testimony|Gilead Sciences, Inc.: Advisor/Consultant|Gilead Sciences, Inc.: Expert Testimony|GSK: Advisor/Consultant|GSK: Expert Testimony|Hepatera: Advisor/Consultant|Hepatera: Expert Testimony|Merck Sharp & Dohme: Advisor/Consultant|Merck Sharp & Dohme: Expert Testimony|Roche: Advisor/Consultant|Roche: Expert Testimony|R-Pharm: Advisor/Consultant|R-Pharm: Expert Testimony Grace Chee, PharmD, Gilead Sciences, Inc.: Employee|Gilead Sciences, Inc.: Stocks/Bonds (Private Company) Dmitry Manuilov, MD, Gilead Sciences, Inc.: Employee|Gilead Sciences, Inc.: Stocks/Bonds (Private Company) Mingyang Li, PhD, Gilead Sciences, Inc.: Employee|Gilead Sciences, Inc.: Stocks/Bonds (Private Company) Audrey Lau, MD, PhD, Gilead Sciences, Inc.: Employee|Gilead Sciences, Inc.: Stocks/Bonds (Private Company) Anu Osinusi, MD, Gilead Sciences, Inc.: Employee|Gilead Sciences, Inc.: Stocks/Bonds (Public Company) Steve Tseng, MD, Gilead Sciences, Inc.: Employee|Gilead Sciences, Inc.: Stocks/Bonds (Private Company) Julian Schulze Zur Wiesch, MD, PhD, Gilead Sciences, Inc.: Consultation and lecture fees Markus Cornberg, MD, PhD, AbbVie: Honoraria|Falk: Honoraria|Gilead Sciences, Inc.: Honoraria|GSK: Honoraria|Janssen-Cilag: Honoraria|Merck Sharp & Dohme: Honoraria|Novartis: Honoraria|Roche: Honoraria|Spring Bank Pharmaceuticals: Honoraria|Swedish Orphan Biovitrum: Honoraria Stefan Zeuzem, MD, PhD, AbbVie: Advisor/Consultant|AbbVie: speaker bureau|Allergan: Advisor/Consultant|Allergan: speaker bureau|BioMarin: Advisor/Consultant|BioMarin: speaker bureau|Gilead Sciences, Inc.: Advisor/Consultant|Gilead Sciences, Inc.: speaker bureau|Intercept: Advisor/Consultant|Intercept: speaker bureau|Janssen: Advisor/Consultant|Janssen: speaker bureau|Merck Sharp & Dohme: Advisor/Consultant|Merck Sharp & Dohme: speaker bureau|Novo Nordisk: Advisor/Consultant|Novo Nordisk: speaker bureau|Swedish Orphan Biovitrum: Advisor/Consultant|Swedish Orphan Biovitrum: speaker bureau|Theratechnologies: Advisor/Consultant|Theratechnologies: speaker bureau Pietro Lampertico, MD, AbbVie: speaking and teaching fees/ advisory committees and review panels|Aligos: speaking and teaching fees/ advisory committees and review panels|Alnylam: speaking and teaching fees/ advisory committees and review panels|Antios: speaking and teaching fees/ advisory committees and review panels|Arrowhead: speaking and teaching fees/ advisory committees and review panels|Bristol Myers Squibb: speaking and teaching fees/ advisory committees and review panels|Eiger Pharmaceuticals: speaking and teaching fees/ advisory committees and review panels|Gilead Sciences, Inc.: speaking and teaching fees/ advisory committees and review panels|GSK: speaking and teaching fees/ advisory committees and review panels|Janssen: speaking and teaching fees/ advisory committees and review panels|Merck Sharp & Dohme: speaking and teaching fees/ advisory committees and review panels|MYR GmbH: speaking and teaching fees/ advisory committees and review panels|Roche: speaking and teaching fees/ advisory committees and review panels|Spring Bank Pharmaceuticals: speaking and teaching fees/ advisory committees and review panels

